# Homœopathy, &c. at a Discount

**Published:** 1844-10-19

**Authors:** 


HOMŒOPATHY, &C. AT A DISCOUNT.

A new reformer in Medicine, Ernest Manher by name, has sprung up in Leipsic. The prophet is foreshadowed even in his external appearance. He pretends that he has discovered, or at least brought to light again, the original and primitive Hygiêne; and he has already committed to print a portion of his grand panacea. Abstracting himself from all former medical theories, he rejects alike allopathy and homœopathy; the nearest thing he comes to is hydropathy. It is his innate conviction, that by the adoption of his purely "Natural System," physic and physicians will henceforth be superfluous. That the man possesses a clear understanding, some wit, and is a ready speaker, we cannot deny; he lectures to crowds of students in *convictorio*, and at times also in the Rosenthal. If he is to be believed, he has made a personal trial of his doctrines; for a time he dwelt in the wilds of America; at another, for the purpose of strengthening his system, he practised daily swimming in the sea in the month of Janurary, during a sojourn at Calais; he also took long walks, stark naked, during a keen frost, &c., and all this for the purpose of testing to the utmost the powers of resistance of nature. In a word, his system may be described as emphatically "a hardening one."—*Alg. Zeitung für Chirurgie, &c.*

